# Benzoyl Peroxide Inhibits Quorum Sensing and Biofilm Formation by* Gardnerella vaginalis* 14018

**DOI:** 10.1155/2018/1426109

**Published:** 2018-07-02

**Authors:** Ammar Algburi, Saskia Zehm, Victoria Netrebov, Richard Weeks, Konstantin Zubovskiy, Michael L. Chikindas

**Affiliations:** ^1^Department of Biochemistry and Microbiology, Rutgers State University, New Brunswick, NJ, USA; ^2^Department of Biology and Biotechnology, College of Sciences, Diyala University, Baqubah, Iraq; ^3^Department of Life Sciences and Technology, Beuth University of Applied Sciences, Berlin, Germany; ^4^Health Promoting Natural Laboratory, School of Environmental and Biological Sciences, Rutgers State University, New Brunswick, NJ, USA; ^5^Scientelle, LLC, Morristown, NJ 07960, USA

## Abstract

Infection recurrence and antibiotic resistance of bacterial vaginosis-associated pathogenic biofilms underline the need for novel and effective treatment strategies. In this study, we evaluated the antimicrobial, antibiofilm, and quorum sensing inhibitory effects of benzoyl peroxide and salicylic acid against* Gardnerella vaginalis *ATCC 14018, the predominant pathogen of bacterial vaginosis. While the highest tested concentrations of 250 and 125 *μ*g/mL for both compounds were not sufficient in completely inhibiting the growth of* G. vaginalis* ATCC 14018, they did prevent biofilm formation by inhibiting the bacterial quorum sensing system in the pathogen. To our knowledge, this report is the first evidence that benzoyl peroxide can have a quorum sensing-mediated biofilm controlling effect, as demonstrated using subinhibitory concentrations of this compound in order to reduce the cost, dosage, and negative side effects associated with current antimicrobial treatments.

## 1. Introduction

The challenge to treat bacterial vaginosis (BV), the most common polymicrobial infection in women of reproductive age [1], is that it is often associated with infection recurrence after initial antibacterial treatment [2]. Multiple studies published between 2010 and 2015 reported a recurrence rate in excess of 50% [3]. BV occurs as a result of an imbalance of the microbial community, where the heathy lactobacilli microbiota is suppressed by BV-associated microorganisms, with* G. vaginalis* playing one of the central roles [1]. The etiology of BV is still controversial, though it is likely that various microorganisms and conditions contribute to the development and persistence of BV.* G. vaginalis *is of particular significance in the study of BV, as it represents a potentially pathogenic microorganism that, while present among the commensal vaginal microbiota, is also thought to be an essential component in the initiation and propagation of BV [4]. BV occurrence and recurrence depend on the development of a multispecies biofilm with* G. vaginalis* as the dominant species among a diversity of other BV-associated pathogens that are present in varying numbers [5, 6]. The formation and presence of single or multispecies biofilms are common features among many persistent infections [7]. Biofilm formation begins when free floating planktonic bacteria reach a certain cellular density, initiating quorum sensing (QS) triggered changes in the levels of expression of fimbriae, flagella, and so forth, which allow the organism to be more easily attached to the surface. Once attached, the bacteria undergo several changes in the expression of key biofilm formation genes, and the bacteria become attached to the colonized surface, adopting a sessile lifestyle. Once sessile, the bacteria undergo additional changes, producing a network of extracellular polymeric substances (EPS) and so forth. Initial attachment and subsequent changes are driven by QS. QS is a phenomenon in which various signaling molecules released into the extracellular environment regulate the expression of genes across the entire community of this biofilm's associated microorganisms; this process is concentration-dependent [8, 9]. It has been suggested by Hardy et al. [6] that* G. vaginalis* may serve as the initial anchor by which this polymicrobial biofilm is initiated in BV, an assumption that is supported by* G. vaginalis' *greater virulence potential, as compared to other BV-associated pathogens. Moreover, several experiments have shown increased* G. vaginalis *biofilm mass when cocultured with selected BV pathogens [4].* G. vaginalis *biofilms are more resistant to conventional antimicrobial treatments, likely due to the matrix of extracellular polymeric substances that form a barrier between the bacteria and the lumen, strong attachment to the epithelia surface, and positive interactions among the species within the biofilm itself. [6]. Swidsinski et al. reported that* G. vaginalis* biofilms were only temporarily suppressed during metronidazole treatment and, in most cases, rapidly regained activity following treatment cessation [10]. As such,* G. vaginalis* remains the primary pathogen of interest in the occurrence and recurrence of bacterial vaginosis.

In the QS phenomenon, multifunctional signaling molecules known as autoinducers (AIs) can regulate the gene expression of microbes and switch their lifestyle from planktonic to sessile communities [11]. It is important to note that quorum sensing differs between Gram-positive and Gram-negative organisms, with different signaling molecules, receptors, and associated regulatory pathways in each instance [8, 9].

Previously, we reported on the possible role of QS and AIs in biofilm formation by* G. vaginalis* and studied* in vitro* the role of QS modification with exogenous agent in a context of potential treatment of BV or prevention of its recurrence [12]. Should such an agent be able to inhibit the QS communication involved in triggering pathogen virulence and biofilm formation, it could potentially be utilized to directly modify BV-associated bacteria and consequently treat and prevent BV. It has also been suggested that the use of QS inhibitors may reduce the risk of developed resistance, as they act as antivirulence agents as opposed to traditional bacteriostatic and bactericidal compounds [13]. In addition, the deterioration of biofilms through targeting QS could increase the effectiveness of available BV-targeted drugs.

In this work, we evaluated the QS inhibitory effect of two compounds, benzoyl peroxide (BP) and salicylic acid (SA), in preventing the formation of* G. vaginalis* biofilms. BP, an organic peroxide compound, is listed in the “World Health Organization's List of Essential Medicines” as an essential and basic antimicrobial agent required for human health [14]. The activity of BP is mostly related to the production of reactive oxygen species (ROS) [15].

SA, a phenolic metabolite found in plant extracts, has shown antibiofilm activity against Gram-positive and Gram-negative bacteria [16, 17]. It has been reported that SA inhibits the production of teichoic acid and slime-associated proteins in wild and polysaccharide/adhesins-deficient mutant strains of* Staphylococcus epidermidis *[16]. In addition, SA may be used for coating medical devices as a film-releasing polymer to reduce the formation of pathogenic biofilms such as* Escherichia coli* in the urinary tract [18]. Prevention of biofilm formation by SA causes an indiscriminate change in cell density while simultaneously targeting and blocking the production of QS-signaling peptides, such as acyl homoserine lactones (AHLs), by inhibiting QS-regulated genes expression in* Pseudomonas aeruginosa* [19, 20]. SA also has a suppressive effect on bacterial flagella, which significantly reduces swarming motility and subsequently leads to a reduction in biofilm formation [21].

This report evaluates the effect of two compounds, BP and SA, as QS inhibitors in* G. vaginalis* 14018.* Chromobacterium violaceum *ATCC 12472 (biological method linked to pigment production) was used as a microbial reporter to identify QS inhibition in Gram-negative bacteria, while an Fe (III) reduction method (chemical assay) was used for Gram-positive bacteria. This study is likely to be the first report on BP-driven inhibition of QS and biofilm formation.

## 2. Materials and Methods

### 2.1. Bacterial Strains and Growth Conditions

*Gardnerella vaginalis* ATCC 14018 strain was grown in Brain-Heart Infusion (BHI) medium (Difco, Sparks, MD) supplemented with 3% of horse serum (sBHI) (JRH Biosciences, KS) and incubated at 37°C overnight and anaerobically (10% hydrogen, 5% carbon dioxide, and 85% nitrogen) using the anaerobic glove box (Coy Laboratory Products, Inc., Grass Lake, MI). BHI medium supplemented with 1% glucose (BHIG) was used in biofilm formation assays.* C. violaceum* ATCC 12472 was grown in Luria-Bertani (LB) broth (ACROS, Miller, NJ) at 26°C for 48 h aerobically.* C. violaceum* ATCC 12472 was used as a bacterial reporter for the QS inhibition assay for Gram-negative bacteria in which* P. aeruginosa* ATCC 14213, the positive control, was grown aerobically in LB broth at 37°C for 24 h. As representatives of Gram-positive and Gram-negative bacteria,* Listeria monocytogenes* Scott A and* E. coli *O157:H7, respectively, were grown in sBHI and incubated aerobically for 18-24 h at 37°C.

### 2.2. Chemicals and Antimicrobial Compounds

In this study, the chemicals used were hydrous benzoyl peroxide (Spectrum Chemical Mfg. Corp., Gardena, CA), salicylic acid (Sigma Aldrich, Milwaukee, WI), L-ascorbic acid (Sigma Chemical Company, St. Louis, MO), and dimethyl sulfoxide (DMSO) (Fisher Scientific, Fair Lawn, NJ). The working solution for detection of QS signals (AI-2) in Gram-positive bacteria was prepared according to Wattanavanitchakorn et al. [22]. Briefly, 0.198 g of 1,10-phenanthroline was dissolved in 50 mL of double distilled water (ddH_2_O) and the pH of the solution was adjusted to 2.0 using 1 M HCl. Ferric ammonium sulphate (0.16 g) was mixed with the solution and the volume was completed to 100 mL by adding ddH_2_O. The final concentration of the 1,10-phenanthroline/3.32 mM Fe(III) working solution was prepared to 10 mM.

### 2.3. Minimum and Sub-Minimum Inhibitory Concentration (MIC and Sub-MIC)

Sub-MICs are concentrations below MIC values and of those selected for the study are the one(s) that do not cause visible inhibition of microbial growth as judged by the kinetics of the measurement of the OD at 595-600 nm [7]. To determine the MIC and sub-MIC of BP and SA, a broth microdilution assay was performed following Algburi et al. [23]. Briefly, aliquots of the overnight growth of bacterial strains (*L. monocytogenes* Scott A,* E. coli *O157:H7, and* G. vaginalis* 14018) were diluted 1:100 (*v:v*) into fresh sBHI to achieve about 10^6^ CFU/mL. The bacterial cell numbers (CFU/mL) were determined using spot plate method. To prepare the SA, 10 mg of SA was dissolved in 20 mL sBHI to have a concentration of 500 *μ*g/mL and sterilized under UV for 20-25 min. In addition, BP was prepared by dissolving 10 mg of BP in 200 *μ*L of DMSO; the volume was then completed to 20 mL with sterile sBHI to have 500 *μ*g/mL of BP. Once the solutions were prepared, 100 *μ*L samples of BP and SA were transferred in triplicates and serially twofold diluted with sBHI into a 96-well tissue culture microplate (Falcon, Corning Inc., Corning, NY). A 100 *μ*L aliquot of the bacterial suspension (10^6^ CFU/mL) was added to each well in the 96-well microplate treated with different concentrations of BP and SA. Positive (bacterial cells into broth) and negative controls (broth only, broth with antimicrobials) were included in this assay. To avoid evaporation of contents during the overnight incubation, a 75 *μ*L aliquot of mineral oil (Sigma-Aldrich chemical, St. Louis, MO) was added to each treated well. After incubation, a statistical analysis of the kinetic readings of bacterial growth was performed in order to determine the MICs and sub-MICs of both BP and SA. Aerobic conditions for 18-24 h at 37°C were provided for* L. monocytogenes* Scott A and* E. coli* O157:H7, while anaerobic conditions for 24-36 h at 37°C were provided for* G. vaginalis* 14018.

### 2.4. Biofilm Inhibition Assay

The biofilm inhibition assay was performed similar to the broth microdilution assay with some exceptions. Following Toole [24] with minor modifications, aliquots of overnight growth of bacterial strains were diluted into fresh medium, BHIG used for* G. vaginalis* 14018 and sBHI for* E. coli *O157:H7, to achieve approximately 10^6^ CFU/mL. The antimicrobials were prepared (as mentioned in the MIC assay) and serially twofold diluted with the appropriate culture medium into a 96-well tissue culture microplate. Once the 96-well microplate was prepared, a 100 *μ*L aliquot of the bacterial suspension (10^6^ CFU/mL) was added to each well. A sealing tape (Thermo Scientific, Rochester, NY, USA) was applied onto the wells to avoid evaporation of the sample after overnight incubation. The microplate was incubated for 36-48 h at 37°C without agitation. After incubation, the unattached cells were aspirated by careful pipetting; then each well was gently washed twice with 100 *μ*L of fresh culture medium. Both of the aspirated and washable (planktonic) cells were collected and diluted (10^1^-10^7^) for counting CFU/mL using Spot Plate Method [23]. After washing, the biofilm's biomass was quantified according to Borucki et al. [25] with minor modifications. Briefly, the intact biofilm was fixed at 60°C for 60 minutes in an inverted position. To quantify the biofilm, 125 *μ*L of a 0.1% solution of crystal violet (CV) in water was added to each treated well of the microplate. Then, the microplate was incubated at room temperature for 15-20 min. After the incubation period, 200 *μ*L of sterile water was used to rinse each well of the microplate 3-4 times. After rinsing the wells, 200 *μ*L of 95% ethanol was added to the wells to solubilize the CV and the plate was incubated at 4°C for 30 min. Following incubation, 100 *μ*L of solubilized CV was transferred into a new flat bottomed 96-well microplate. The absorbance of each well was recorded using a plate reader at optical density of 595 nm (Model 550, Bio-Rad Laboratories, Hercules, CA).

### 2.5. QS Inhibition Assay in Gram-Negative Bacteria

The overnight growth of* C. violaceum* ATCC 12472 in LB broth was diluted in fresh medium to achieve 10^6^ CFU/mL. Each antimicrobial was serially twofold diluted with LB into a 48-well microplate (BD, Franklin lakes, NJ), starting with 1X MICs and including sub-MICs, the concentrations that did not influence the growth of planktonic cells. Once the antimicrobial was diluted, a 500 *μ*L aliquot was added to each well and a 500 *μ*L aliquot of bacterial growth dilution (10^6^ CFU/mL) was added and mixed with the antimicrobial to achieve a total volume of 1 mL. The mixtures of antimicrobials and cells were aerobically incubated at 26°C without shaking for 48 h. The cell-free supernatant (CFS) of* P. aeruginosa* was used as the positive control, preventing or antagonizing violacein's production of* C. violaceum *ATCC 12472. After incubation, 750 *μ*L of each well (antimicrobials and bacterial cells) was transferred to a 1 mL tube and centrifuged at 8000*g* for 5 min in order to precipitate the violacein. The supernatants were discarded and the pellets were vigorously vortexed with 750 *μ*L of 100% DMSO to ensure that the insoluble violacein was dissolved. The contents were centrifuged again at 8000*g* for 5 min in order to precipitate the* C. violaceum* ATCC 12472 cells. For quantification of violacein production, 200 *μ*L of violacein-containing supernatants of each tube was transferred into a non-tissue culture 96-well microplate (Fisherbrand, USA) in quadruplicates. Quantification of violacein was measured using a microplate reader at a wavelength of 585 nm. To confirm that it was violacein production inhibition but not bacterial growth inhibition, the precipitated* C. violaceum* cells were resuspended in 750 *μ*L distilled water and their turbidity was measured using the plate reader at the optical density of 595 nm. Turbidity of antimicrobials-treated cells was compared with the positive control.

### 2.6. QS Inhibition Assay in Gram-Positive Bacteria

This assay was performed following Wattanavanitchakorn et al. [22] with minor modifications. Briefly, the bacterial species used in this assay included* G. vaginalis* 14018 (tested microorganism) and* L. monocytogenes* Scott A as a positive control (AI-2^+^). In addition,* E. coli* O157:H7 was used a representative Gram-negative pathogen reported as having its biofilm formation influence by AI-2 [26]. The bacterial species were inoculated into their suitable culture media and incubated for 18-24 h at 37°C. After the incubation period, the overnight grown bacteria were diluted in fresh sBHI broth to achieve 10^6^ CFU/mL. AI-2 production was measured after 0, 3, 5, 7, 11, 16, and 24 h of incubation in order to determine the time point at which the highest QS signals are produced. Each antimicrobial, BP and SA, was prepared to a final concentration of 250 *μ*g/mL and 125 *μ*g/mL in sBHI containing 10^6^ CFU/mL of bacterial cells. After 18-24 h incubation at 37°C, the bacterial species were centrifuged (8000*g* for 10 min) and 1 mL of CFS was mixed with 1 mL of working solution (mentioned under “Chemicals and Antimicrobial Compounds”) and left at room temperature for 15 min. The volume of mixture was completed to 5 mL by adding 3 mL of ddH_2_O and centrifuged again (8000*g* for 5 min). After centrifugation, 200 *μ*L aliquots were transferred to a non-tissue culture 96-well microplate (Falcon, Corning Inc., NY, USA) and the optical density (OD) was measured at 510 nm using a microplate reader (ThermoMax, Molecular Devices, USA). The OD reading of treated samples was compared with the controls in order to evaluate QS inhibition by BP and SA.

### 2.7. Statistical Analysis

All experiments were conducted thrice in triplicate. After biofilm staining with crystal violate, the percentages of biofilm inhibition were determined by comparing the absorbance of antimicrobial-treated biofilm to untreated biofilm (the positive control) using plate reader at 595 nm [12]. The standard deviations in each figure are represented by error bars. All the statistical analyses were conducted in Microsoft Excel and graphed with SigmaPlot 11.0 (Systat Software Inc., Chicago, IL, USA).

## 3. Results

### 3.1. Determination of MICs and Sub-MICs

**Figure 1:**
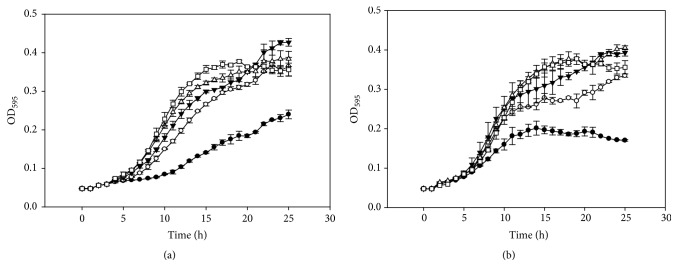
Benzoyl peroxide (a) and salicylic acid (b) activity against* G. vaginalis*. Benzoyl peroxide 250 *µ*g/mL (●), 125 *µ*g/mL (○), 62.5 *µ*g/mL (▼), 31.3 *µ*g/mL (Δ), and 0 *µ*g/mL (□). Salicylic acid 250 *µ*g/mL (●), 125 *µ*g/mL (○), 62.5 *µ*g/mL (▼), 31.3 *µ*g/mL (Δ), and 0 *µ*g/mL (□).

**Figure 2:**
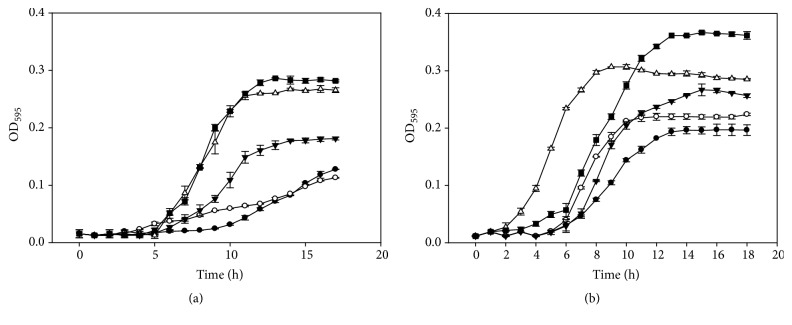
Benzoyl peroxide (a) and salicylic acid (b) activity against* L. monocytogenes*. Benzoyl peroxide 250 *µ*g/mL (●), 125 *µ*g/mL (○), 62.5 *µ*g/mL (▼), 31.3 *µ*g/mL (Δ), and 0 *µ*g/mL (■). Salicylic acid 250 *µ*g/mL (●), 125 *µ*g/mL (○), 62.5 *µ*g/mL (▼), 31.3 *µ*g/mL (Δ), and 0 *µ*g/mL (■).

**Figure 3:**
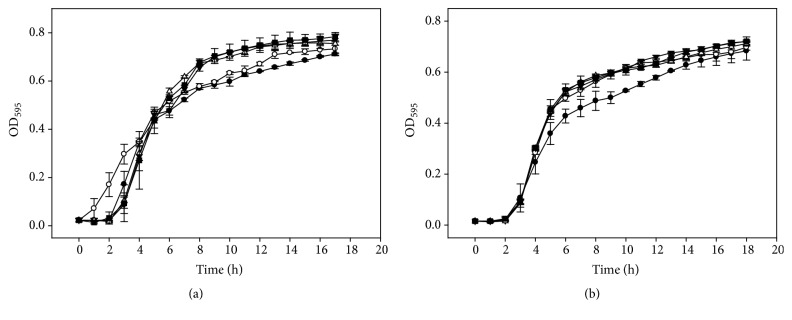
Benzoyl peroxide (a) and salicylic acid (b) activity against* E. coli*. Benzoyl peroxide 250 *µ*g/mL (●), 125 *µ*g/mL (○), 62.5 *µ*g/mL (▼), 31.3 *µ*g/mL (Δ), and 0 *µ*g/mL (■). Salicylic acid 250 *µ*g/mL (●), 125 *µ*g/mL (○), 62.5 *µ*g/mL (▼), 31.3 *µ*g/mL (Δ), and 0 *µ*g/mL (■).

A broth microdilution assay was used to determine MICs and sub-MICs of BP and SA against the tested pathogens. A concentration greater than 250 *μ*g/mL of BP and SA was required to completely inhibit the growth of* G. vaginalis* ATCC 14018. The sub-MICs for both compounds were determined to be 250 and 125 *μ*g/mL, respectively (Figure 1). These two concentrations were used in the biofilm inhibition assay, especially 125 *μ*g/mL, since it did not influence the growth of* G. vaginalis*. For* L. monocytogenes* Scott A and* E. coli *O157:H7, more than 250 *μ*g/mL of BP and SA was needed to completely inhibit the growth of bacterial cells. The sub-MICs of the antimicrobials ranged from 31.3 to 250 *μ*g/mL with slight inhibitory effects on* L. monocytogenes* Scott A growth, while* E. coli *O147:H7 was tolerant to concentrations up to 250 *μ*g/mL of BP (Figures 2 and 3).

### 3.2. Biofilm Inhibition by BP and SA

**Figure 4:**
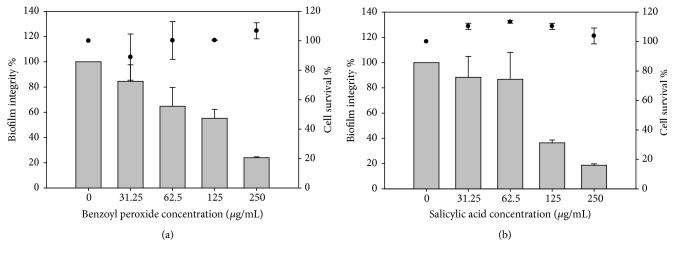
Inhibition of* G. vaginalis* biofilm by benzoyl peroxide (a) and salicylic acid (b). Biofilm integrity % (gray colour); cell survival % (●).

Crystal violet (CV), as a colorimetric method, was used for biofilm staining and to determine the biofilm quantity after treatment in order to identify whether biofilm formation was prevented by BP and SA. Approximately 80% of* G. vaginalis* ATCC 14018 biofilm formation was inhibited when the cells were treated with a sub-MIC concentration (250 *μ*g/mL) of BP and SA, as compared to the control. There was 50% and more than 40% of biofilm prevention at a concentration of 125 *μ*g/mL of BP and SA, respectively, as compared to the control. Additionally, the viability of the bacterial cells was not influenced by BP and SA, even when a high concentration, 250 *μ*g/mL, was used (Figures 4(a) and 4(b)). It is unclear whether these concentrations inhibit the quorum sensing system of* G. vaginalis* ATCC 14018 or impede their attachment to the microplate surface.

**Figure 5:**
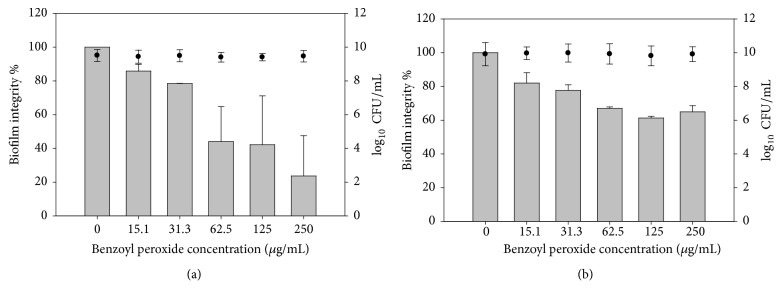
Inhibition of* L. monocytogenes* (a) and* E.coli* (b) biofilm by benzoyl peroxide. Biofilm integrity (gray colour); cell survival (●).

BP was more active in preventing* L. monocytogenes *Scott A biofilm formation as compared to* E. coli *O157:H7 biofilm. Our results in Figure 5(a) showed that about 80% and 60% of* L. monocytogenes* Scott A biofilm were inhibited when 125 and 250 *μ*g/mL of BP were used, respectively. Only 30-35% of* E. coli* O157:H7 biofilm was reduced when 125 or 250 *μ*g/mL of BP was applied (Figure 5(b)). Also, the normal growth ability of bacterial cells was not affected, even when high concentrations of both substances were used.

### 3.3. BP and SA Inhibited Violacein Production but Not Bacterial Growth of* C. violaceum *ATCC 12472

**Figure 6:**
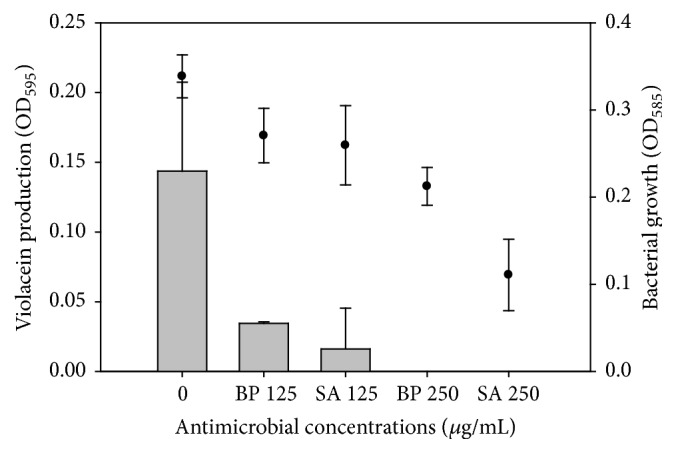
Effect of benzoyl peroxide (BP) and salicylic acid (SA) on violacein production and growth of* C. violaceum.* Violacein production (OD_595_) (gray colour); bacterial growth (OD_585_) (●).

In comparison to untreated cells, 250 *μ*g/mL of both BP and SA completely prevented violacein production with slight inhibition in the growth of* C. violaceum* ATCC 12472. More than 80% of violacein production was inhibited without influencing the growth of* C. violaceum* ATCC 12472 when 125 *μ*g/mL of both BP and SA was applied (Figure 6). The growth of* C. violaceum* ATCC 12472, like in* G. vaginalis *14018, was slightly influenced by 125 *μ*g/mL of BP and SA, indicating a possible quorum sensing inhibitory effect of both substances.

### 3.4. Inhibition of AI-2 Production in the Presence of BP

**Figure 7:**
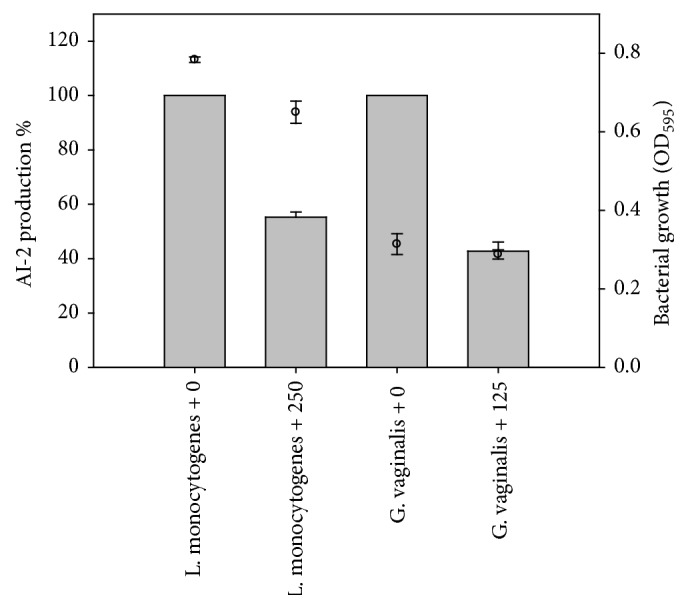
Inhibition of AI-2 production by* L. monocytogenes* and* G. vaginalis* in the presence of 250 and 125 *µ*g/mL of benzoyl peroxide. AI-2 production % (gray colour); bacterial growth (OD_585_) (●).

In the presence of BP, AI-2 production by* G. vaginalis *14018 and* L. monocytogenes *Scott A was inhibited. The production of AI-2 by* G. vaginalis* 14018 was reduced by more than 50% in the presence of 125 *μ*g/mL of BP without effecting the bacterial growth when compared to untreated bacterial cells (Figure 7). At 250 *μ*g/mL of BP, AI-2 production was fully inhibited and bacterial growth of* G. vaginalis* 14018 was partially suppressed (data not shown). Similarly, AI-2 production by* L. monocytogenes *Scott A was reduced to 50% when treated with BP at a concentration of 250 *μ*g/mL, with little inhibition in bacterial growth compared to the control (Figure 7).

## 4. Discussion

The imbalance of vaginal microbiota in cases of bacterial vaginosis (BV) is associated with dysbiosis of the normal vaginal flora, with a loss of* Lactobacillus* species [27] and increased growth of a number of other anaerobic species that may or may not be present in the healthy vaginal environment, predominantly* G. vaginalis *[28]. The central role of* G. vaginalis* in BV is attributed to the pathogen's virulence factors and the highest propensity to form biofilm among BV-associated bacteria [27–29]. In biofilm,* G. vaginalis* has high tolerance to antimicrobials, which creates additional clinical challenges [30]. It is plausible to assume that very high reported rates of recurrence of BV may be associated, at least in part, with the biofilm-forming potential of* G. vaginalis*. Therefore, it becomes important to evaluate antimicrobials with a potential for BV treatment on their ability to arrest* G. vaginalis* virulence and its biofilm-forming capacity.

SA's potential for inhibiting biofilm formation in pathogenic bacteria has been reported in several studies [21, 31]. In* P. aeruginosa,* SA has been shown to cause a reduction in bacterial swarming movement, rather than twitching and swimming motility, leading to inhibition of biofilm formation [21]. However, previous studies have reported that SA does not affect housekeeping genes; therefore biofilm formation inhibition or motility inhibition does not interrupt critical cellular processes necessary for survival [32, 33]. In addition, SA has been reported to inhibit the production of teichoic acid and slime-associated proteins in wild and polysaccharide/adhesins-deficient mutant strains of* Staphylococcus epidermidis *[16]. In agreement, it has been noticed that SA inhibits bacterial aggregation and attachment at the air-liquid interface without influencing the bottom-forming films [31].

It has been reported that SA has an anti-QS inhibitory effect. Using* C. violaceum* CV026 as a biosensor for violacein production, Chang et al. [34] found that there was no AHL production when L-arabinose-induced* E. coli* MG1655 cultures were treated with SA as determined using* C. violaceum* CV026, indicating that SA is capable of repressing the QS system and attenuating virulence-associated biofilm formation. However, AHLs were detected by the more sensitive liquid chromatography-mass spectrometry methods. This suggests that the observed QS regulation is concentration-dependent. Bandara et al. [19] also reported repression of AHL production in* P. aeruginosa* when SA was applied using a* C. violaceum* CV026 biosensor, in addition to a reduction of bacterial cytotoxicity against human corneal epithelial cells. In this study, the antimicrobial activity of benzoyl peroxide against* G. vaginalis *14018 was evaluated and it was found that 250 *μ*g/mL (sub-MIC) of BP caused a partial inhibition of microbial growth. Concentrations higher than 250 *μ*g/mL of BP were not tested because of its turbidity when suspended or dissolved in 10% DMSO. SA was as active as BP against* G. vaginalis *14018, and 250 and 125 *μ*g/mL did not completely inhibit the growth of* G. vaginalis *14018. SA and BP, at the above-mentioned concentrations, were able to inhibit biofilm formation with an indiscriminate inhibitory effect on* G. vaginalis* 14018 growth (Figure 4).* L. monocytogenes* Scott A and* E. coli *O157:H7 were also included as controls in this study, as representatives of Gram-positive and Gram-negative species, with BP found to be more effective in inhibiting biofilm formation in* L. monocytogenes* Scott A than in* E. coli *O157:H7. While biofilm formation by* E. coli *O157:H7 was inhibited to a lesser extent than in* L. monocytogenes* Scott A, we cannot exclude the possible effect of partially inhibited AI-2 on the efficiency of biofilm formation by this Gram-negative pathogen [26].

In several studies such as Coenye et al. [35] and Nusbaum et al. [36], the antibacterial potential of BP against bacterial pathogens alone and in combination with antibiotics has been reported. BP showed a strong biocidal effect against* P. acnes,* both fully sensitive and resistant strains [37], suggesting the importance of BP when used in combination with antibiotics against persistent infections. In Ozolins et al.'s work [38], BP showed similar activity when compared to tetracycline and minocycline, making BP a viable choice for cost-effectiveness treatment. In the context of BV treatment, it was reported that a BP formulated polycarbophil/carbopol 934P hydrogel had an inhibitory effect on the growth of* G. vaginalis* with little to no effect on* Lactobacillus* species [39]. Although the bactericidal effect of ROS is known [40], it is possible that some ROS may also influence pathogen-associated biofilms by impacting QS. When decomposed, BP releases free oxygen radicals that disrupt vital cellular components [41]. ROS have also been shown to disrupt the Fe-S cluster synthesis process [42], an essential system in the growth of* P. aeruginosa*. However, other studies indicate that this possible biofilm prevention effect is species-specific and cannot be generalized to other ROS.

With regard to biofilm, Nusbaum et al. [36] reported that 5% BP alone as an antibiofilm agent was not effective, while a significant effect was noticed when it was combined with either erythromycin or clindamycin. The authors claim that* P. acnes* was not vulnerable to BP without the addition of protein synthesis inhibition due to erythromycin or clindamycin. In agreement with this study, a combination of 5% benzoyl peroxide + 0.5% erythromycin and 5% benzoyl peroxide + 1% clindamycin effectively inhibited biofilm formation and produced a 3-log reduction in the number of biofilm-associated* P. acnes* cells [35].

Biofilm inhibition by BP is possibly related to its lipophilic properties, elaboration of benzoic acid, and/or generation of ROS. It is possible that BP can penetrate or disrupt the plasma membrane of* G. vaginalis *due to its lipophilic properties [41, 42], eventually killing bacterial cells due to BP-associated oxidative potential or inhibiting biofilm formation by interrupting bacterial adhesion. Lou et al. [43] reported that benzoic acid, a metabolic byproduct of BP, inhibits biofilm-associated* P. aeruginosa*. Furthermore, ROS inhibit biofilm formation of* E. coli *by disrupting indole signaling, which is increased as a result of high tryptophanase expression [44]. The less tryptophanase (TnaA) production was, the more* E. coli* biofilm was restored.

In this study, we noticed a relationship between biofilm inhibitions in* G. vaginalis* using sub-MIC concentrations of BP linked to quorum sensing inhibition. The antimicrobial activity of BP is associated with ROS, which have shown the potential to influence biofilm formation in several microorganisms. Some studies referred to the ability of ROS to prevent biofilm formation in* S. aureus* by inhibiting autoinducer molecule signaling and in* B. subtilis* by repressing the expression of locus* comQXP-*associated QS [45]. Additionally, it has been shown that S-Ribosylhomocysteine (LuxS), a mononuclear iron protein [46], can be influenced by ROS that target mononuclear iron enzymes, indicating their possible roles in biofilm formation [47].

## 5. Conclusion

In comparison to conventional antibacterial strategies that combine bactericidal and biofilm-removing activities, the possibility of counteracting quorum sensing-mediated biofilm formation is an alternative approach by which dosage, cost, and harmful effects may be reduced. However, it must be noted that the use of anti-QS compounds to control human diseases still requires more studies, and the work presented herein is just the first step towards that goal. QS inhibitors face the usual challenges inherent to drug discovery (toxicity, stability, efficacy, etc.) but present unique challenges of their own. One of the biggest questions is when and how to apply these compounds. As antivirulence factors, they may serve best as prophylactics to prevent initial biofilm formation, or, as is often suggested, in combination treatments with traditional antimicrobials with the goal of preventing recurrence, a common theme in BV and other chronic illnesses [13].

To our knowledge, this is the first report showing the relationship between inhibition of quorum sensing by BP and biofilm prevention in* G. vaginalis*. This investigation served as a pilot study by which the biofilm-inhibitory potential of BP and SA treatments has been shown using a single strain of* G. vaginalis *as a model. Future studies should take into account the diversity of BV-associated pathogens, as well as the diversity among* G. vaginalis *strains, and expand on testing of BP and SA to include the full spectrum of BV pathogens. Finally, this investigation exemplifies a promising approach in the treatment of biofilm-associated infections utilizing anti-QS agents active at sub-MIC concentrations. This approach may be extended to other known and newly identified antimicrobial agents.
